# Foot loading is different in people with and without pincer nails: a case control study

**DOI:** 10.1186/s13047-015-0100-y

**Published:** 2015-08-19

**Authors:** Hitomi Sano, Kaori Shionoya, Rei Ogawa

**Affiliations:** Department of Plastic, Reconstructive and Aesthetic Surgery, Nippon Medical School, 1-1-5 Sendagi, Bunkyo-ku, Tokyo, 113-8603 Japan; Shionoya Orthopedic Clinic, Azasekitori 5, Ueta-chou, Toyohashi-city, Aichi-Prefecture Japan

**Keywords:** Pincer nail, Mechanical force, Ingrown nail, Nail deformity

## Abstract

**Background:**

Recent studies suggest that pincer nails are caused by lack of upward mechanical forces on the toe pad. However, clinically significant pincer nails are also often observed among healthy walkers. It was hypothesized that in these cases, the affected toes do not receive adequate physical stimulation from walking and loading. To test this, the gait characteristics of pincer nail cases were assessed by measuring plantar pressure during walking.

**Methods:**

In total, 12 bilateral pincer nail cases (24 affected feet) and 12 age- and sex-controlled healthy control subjects (24 ft) were enrolled in this prospective case–control study. Plantar pressure during free ambulation in both the barefoot and shod state was assessed using a digital pressure-plate system named S-Plate platform (Medicapteurs Co. France). First toe pressure and the frequencies of peak pressure in the first toe, metatarsal head, or other foot areas were calculated.

**Results:**

In both the barefoot and shod state, the pincer nail group had significantly lower pressure on the first toe than the control group. In both the barefoot and shod state, the peak pressure area was mostly the metatarsal head area in the pincer nail group, whereas it was mostly the first toe area in the control group. Binomial logistic regression analysis revealed that peak pressure area was a significant risk factor for pincer nail development.

**Conclusion:**

Walking behavior appears to contribute to pincer nail development. Pincer nails of walkers could be treated by correcting the walking behaviour so that more pressure is placed on the toe pad.

## Background

Fingernails play a key role in protecting the distal phalanges, enhancing tactile discrimination and performing fine manipulation [1–4]. They are also used for scratching and grooming. Toenails contribute to pedal biomechanics. During daily life, nails are constantly exposed to physical stimuli such as counter pressure and shear force. These mechanical forces may therefore play a marked role in nail configuration and participate in the development of nail deformities.

The term pincer nail was introduced by Cornelius and Shellery in 1968 [5] and is defined as a transverse overcurvature of the nail plate. Another condition is ingrown nail, which is a painful inflammatory condition caused by a nail edge that has usually been rendered sharp by improper trimming [6]. Both conditions accompany each other at high rates. Notably, although pincer and ingrown nails are common diseases, their precise etiologies remain unclear. Although poorly fitting shoes have been considered to be one of the most common causes of pincer nails, our previous study showed that bedridden patients who do not wear shoes or bear their own weight have a high incidence of pincer nails [7]. Furthermore, we showed that the nails of fingers that are subject to little physical stimulation tend to curve inward while the nails of fingers that are subject to a lot of physical stimulation tend to have a flat shape [7–9]. While it is generally believed that nail shrinkage is an abnormal phenomenon [6], cumulative research at our hospital has led us to hypothesize that human nails are constitutively equipped with an automatic shrinkage function that allows them to adapt to daily upward mechanical forces. In line with this, we proposed that a pincer nail may be caused by the lack of appropriate mechanical forces on the nail [10]. At face value, the fact that pincer nails are also often observed among healthy walkers could be seen to contradict the latter hypothesis. However, we speculated that, in these cases, insufficient physical stimulation of the toe pad during walking or loading may be the causative factor. To test this hypothesis, the present study on the gait characteristics of pincer nail cases was performed. For this, the plantar pressure during walking was measured.

## Methods

In total, 24 ft of 12 adult participants with bilateral pincer nails and 24 ft of 12 healthy age- and sex-matched participants were enrolled in this prospective case–control study (Table 1). All subjects were free of any ankle and foot pathology. Only participants with pincer nail who were free from any pain after treatment with an super elastic wire, an elastic wire used widely in Japan for conservative pincer nail correction, were eligible for this study [11]. The pincer nail group was compared to the control group for nail configuration and plantar pressure. Furthermore, binomial logistic regression analysis was applied to identify the risk factors for pincer nail development.

**Table 1:** Demographic characteristics of the study participants

	Pincer nail (*n* = 12 cases)	Control (*n* = 12 subjects)	*P* value
Gender	6 females, 6 males	6 females, 6 males	1
Age (years)	48.5 ± 17.8	46.8 ± 11.9	0.7
Height (cm)	163.8 ± 9.2	165.2 ± 7.9	0.58
Weight (kg)	61.3 ± 15.3	61.9 ± 10.3	0.86
BMI (kg/m^2^)	22.6 ± 3.8	22.6 ± 2.7	0.98

The *p* values were generated by Mann-Whitney U tests

The control consisted of healthy age- and sex-matched participants

*BMI* body mass index

### Informed consent

Informed consent was obtained from all individual participants included in the study. All procedures performed in studies involving human participants were in accordance with the ethical standards of the institutional and/or national research committee and with the 1964 Helsinki declaration and its later amendments or comparable ethical standards.

### Measurement of plantar pressure

A digital pressure-plate system named the S-Plate platform (Medicapteurs Co. France) was chosen to measure plantar pressure, because of its portability, accuracy and cost-effectiveness. It was implemented as a device to determine plantar pressure profile after pilot studies confirmed its repeatability. The S-Plate platform can collect data from the right and left foot at the same time during natural walking and is used to measure plantar pressure on both bare and shod feet during free ambulation. The foot was divided into eight anatomical regions: first toe, small toes, medial metatarsal, middle metatarsals, lateral metatarsals, medial arch and lateral arch, as reported by Boyd LA et al. [12]. Three trials were sequentially performed under each condition on the same day. The average pressure of each first toe area was calculated. Moreover, the peak pressure area, which is defined as the site that receives the most intense pressure in the plantar, in each foot and in both in the barefoot and shod state, was determined. Peak pressure is defined as the maximum pressure of the peak pressure area. The frequency with which the first toe, the metatarsal head, or the remaining areas of the foot was the peak pressure area in the barefoot or shod state was calculated for both groups.

### Analysis of first toe nail configuration

**Fig. 1:**
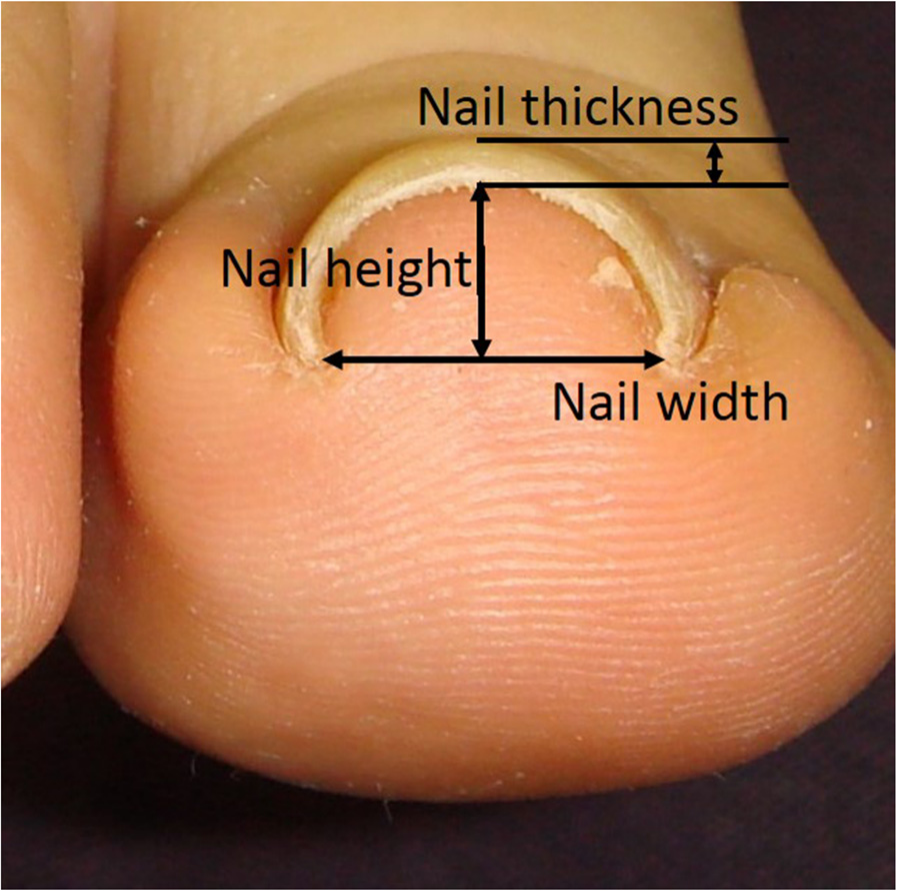
Measurement of nail parameters. The nail height, nail width and the thickness of the central part of the nail plates were measured by using a pair of calipers. The nail width at the distal ends and the nail height were used to quantify the curve index

Nail configuration was measured by calculating the curve index [defined as (nail height/nail width)*100 (%)] and the nail thickness (Fig. 1). The nail height, nail width and the thickness of the central part of the nail plates were measured by using a pair of calipers. For the pincer nail cases, the pre-treatment configuration served as the nail configuration in this analysis.

### Statistical analysis

All data were expressed as means ± SD. Statistical analysis was performed using Microsoft Excel 97–2003 (Microsoft, USA) and SPSS statistical software (SPSS, Chicago, IL). Continuous data were compared by using the Mann-Whitney U test. Categorical data were compared using the chi-square test or Yates chi-square test followed by Haberman residual analysis. Bonferroni’s correction was used to correct for multiple comparisons. The relationship between pincer nail development (the dependent variable) and clinical covariates, including weight, height, BMI, age, sex, peak pressure area (first toe *vs.* other areas of the foot), and the pressure on the first toe were assessed by binomial logistic regression analysis. *p* < 0.05 was considered to indicate statistical significance.

## Results

The Mann-Whitney U test revealed that the pincer nail group had a significantly higher mean first toe nail curve index than the control group (57.9 ± 24.9 % *vs*. 13.9 ± 5.4 %, *p* < 0.001).

**Fig. 2:**
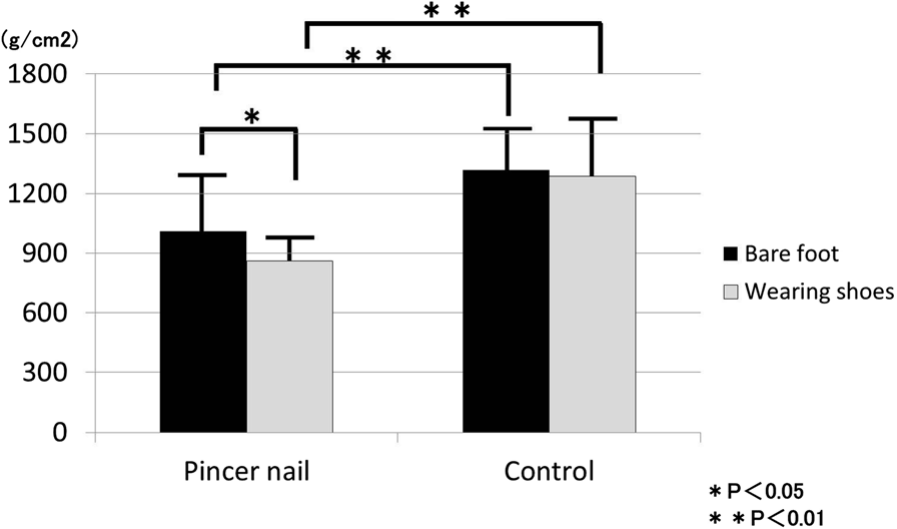
Pressure on the first toes of the pincer nail and control groups. The feet of the pincer nail group (*n* = 24) had significantly less pressure on the first toe than the feet of the control group (*n* = 24). Moreover, in the pincer nail group, the pressure on the first toe was significantly lower when the patients wore shoes than when the patients were barefoot

The Mann-Whitney U test also revealed that the pressure on the first toe of the pincer nail group was significantly lower than that of the control group (bare feet: 1010.0 ± 309.0 *vs.*1318.8 ± 226.2 g/cm^2^, *p* < 0.01, shod fee: 861.7 ± 240.6 *vs.*1287.2 ± 322.9 g/cm^2^, *p* < 0.01). Moreover, in the pincer nail group, the first toe pressure of shod feet was significantly lower than that of bare feet (861.7 ± 240.6 *vs.*1010.0 ± 309.0 g/cm^2^, *p* < 0.05) This difference was not observed in the control group (1287.2 ± 322.9 *vs*1318.8 ± 226.2 g/cm^2^, *p* = 0.71) (Fig. 2).

**Fig. 3:**
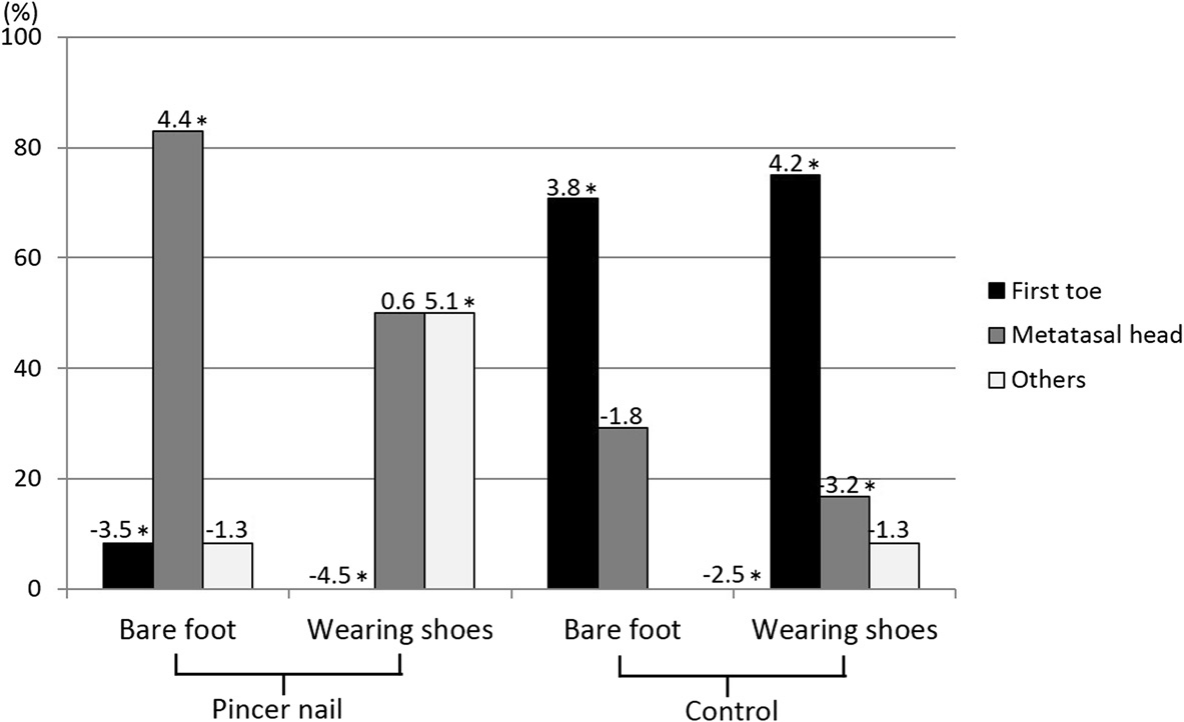
Peak pressure areas of the feet of the pincer nail and control subjects. The metatarsal head area was generally the peak pressure area for the pincer nail group feet (*n* = 24), while the first toe was generally the peak pressure area for the control group feet (*n* = 24). * < 0.05. The standardized residuals are indicated on top of each bar

Yates chi-square analysis revealed that in the barefoot condition, the pincer nail group differed significantly from the control group in both the barefoot and shod conditions for peak pressure area. These differences from the controls were also observed for the pincer nail group in the shod condition. Haberman residual analysis then revealed that in the pincer nail group, the first toe area associated with significantly negative standardized residuals in both the barefoot and shod conditions. By contrast, in the control group, the standardized residuals for the first toe area were significantly positive in both the barefoot and shod conditions. In the pincer nail group, the metatarsal and others areas tended to associate with positive standardized residuals in both the barefoot and shod conditions. By contrast, in the control group, the standardized residuals for the metatarsal and others areas tended to be negative in both the barefoot and shod conditions (Fig. 3). It was notable that the peak pressure area was never the first toe area in the pincer nail feet when the participants were wearing shoes (0/24 ft; 0 %): by contrast, the first toe was the peak pressure area in two of the 24 pincer nail feet when they were bare (8.3 %) and in 17 and 18 of the 24 control feet in the bare and shod conditions (70.8 and 75 %, respectively).

Binomial logistic regression analysis revealed that peak pressure area (*p* = 0.015, odds ratios = 20.8, 95 % confidence intervals 1.82–237.3) was a significant risk factor for pincer nail development.

**Fig. 4:**
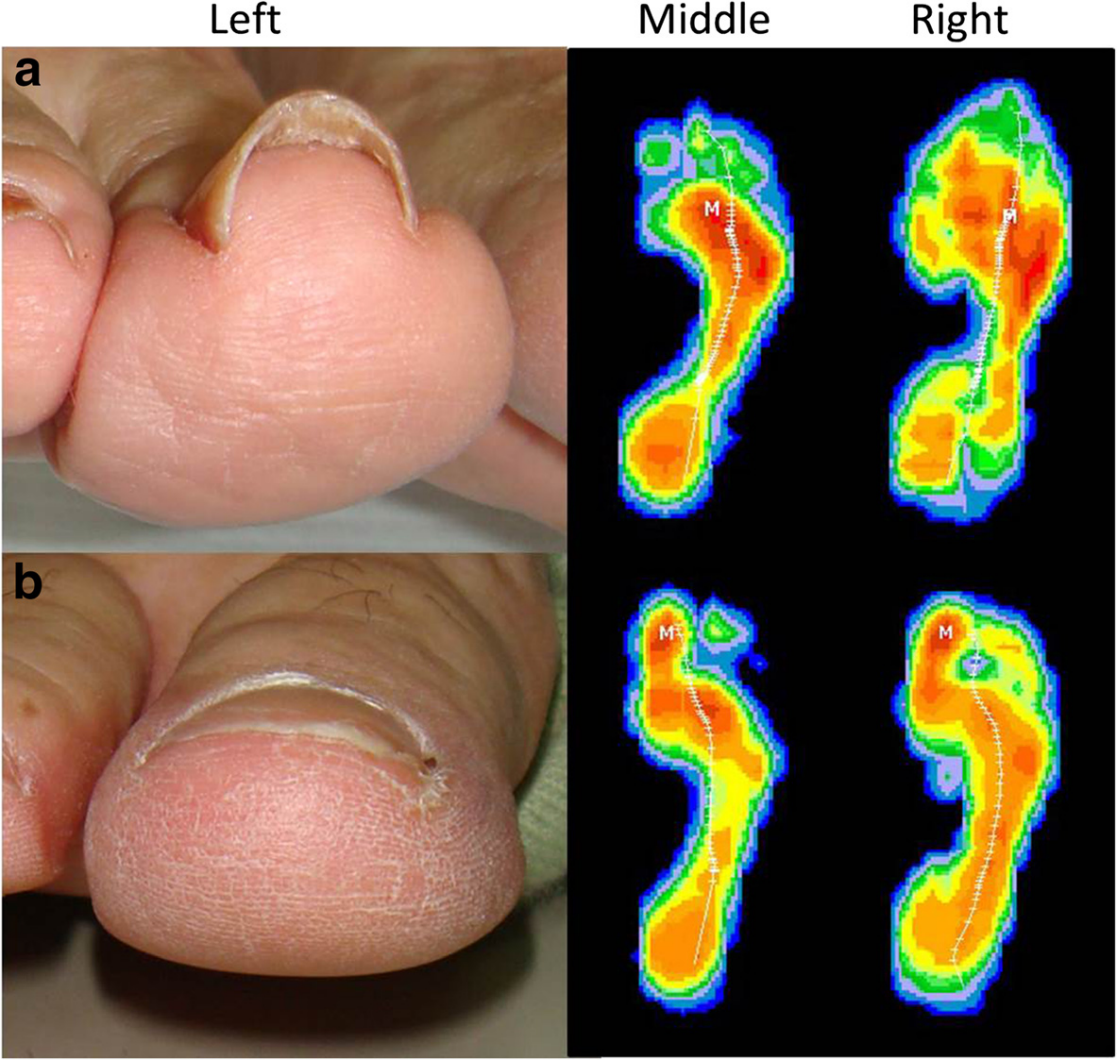
Typical plantar pressure findings of pincer nail cases (**a**) and healthy walkers (**b**). Representative cases are shown. The first toe nail of the pincer nail case (4**a**, *lefthand* image) shows significantly greater inward curvature than the first toe nail of the control case (4**b**, *lefthand* image). The plantar pressures are indicated by a color gradient that ranges from red (very high pressure) to dark blue (very low pressure). The pressure on the first toe in the pincer nail case is much lower than the pressure on the first toe in the control case regardless of whether the feet were bare (*lefthand* foot in panels **a** and **b**) or shod (*righthand* foot in panels **a** and **b**)

Typical plantar pressure findings of pincer nail cases and healthy walkers are shown in Fig. 4. The first toe nail of the representative pincer nail case (Fig. 4a, lefthand image, curve index = 77.8 %) curved inwardly significantly more than the first toe nail of the control case (Fig. 4b, lefthand image, curve index = 17.6 %). The plantar pressures of these pincer nail and control cases in both the barefoot (lefthand foot) and shod (righthand foot) conditions are indicated in the righthand images of Fig. 4a and b by a color gradient that ranged from red (very high pressure) to dark blue (very low pressure). In the pincer nail case, there was weak pressure on the first toe in both conditions whereas the first toe was the peak pressure area in the control case in both conditions.

## Discussion

The nail consists of the nail plate, the nail fold, the nail matrix, the sterile matrix and the hyponychium [13, 14]. The nail configuration is influenced by genetic factors, the shape of the distal phalangeal bone [15], mechanical force [6–8], malnutrition [16], neurogenic factors [17, 18], blood flow [19, 20], and factors that cause the thinning and softening of nails [21]. The causative factors of pincer nail include hereditary, inappropriate physical forces due to ill-fitting shoes, and inappropriate nail cutting [22–26]. Recent researchers have revealed that mechanical forces may have a particularly pronounced effect on nail configuration and may be involved in nail deformity development [7–10]. Namely, pincer nails may be caused by the lack of upward mechanical forces [7]. However, pincer nails are also often observed among healthy walkers. In these cases, the physical stimulation on the toe pad that is delivered by walking and loading may be insufficient. To test this notion, the gait characteristics of patients with a pincer nail were assessed in the present study.

Indeed, plantar pressure analysis of these patients and healthy control participants revealed that the first toes of the pincer nail group experienced significantly less pressure during free ambulation than the first toes of the control group, both in the barefoot and shod conditions. Moreover, compared to the barefoot condition, the pressure on the first toes of the pincer nail group was significantly reduced by wearing shoes. Given that poorly fitting shoes are considered to be a major cause of pincer nails, this observation suggests either that patients with pincer nail tend to wear bigger shoes to avoid pain or that pincer nail develops because the first toe nail does not receive sufficient pressure from the ill-fitting shoes.

**Fig. 5:**
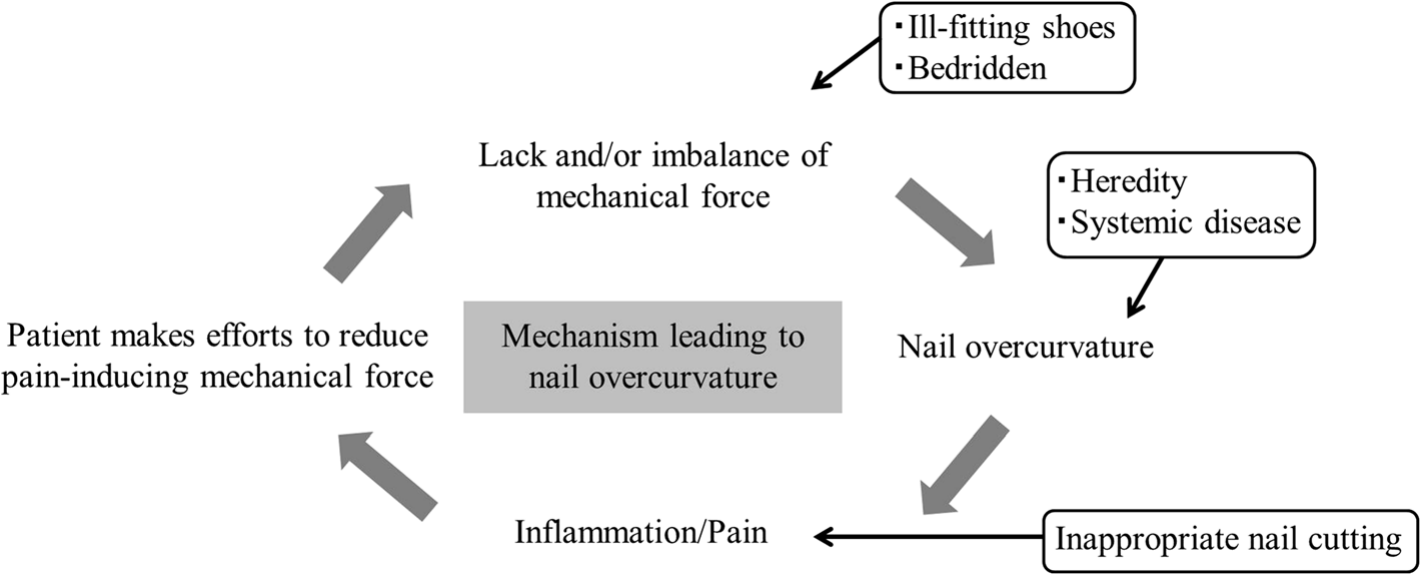
Hypothesis for the development of pincer and ingrown nails. Under normal conditions, the upward mechanical force and the constitutive nail shrinkage force are generally well-balanced and the nail remains normally curved. However, in some people, the constitutive nail shrinkage force may exceed the upward mechanical force, either because the patient lacks sufficient mechanical forces on the nail due to being bedridden or wearing ill-fitting shoes or because the constitutive nail shrinkage force is excessive due to genetic or systemic disease influences. In these cases, the nails curve inward. Inappropriate nail cutting promotes nail overcurvature by inducing inflammation and pain, which causes the patient to seek to reduce pressure on the nail further by reducing ambulation, changing the gait, or wearing ill-fitting shoes. In walkers, this vicious cycle may be abrogated by correcting the gait and shoes

Based on our hypothesis, it seems plausible that the following pathogenic mechanism may be responsible for the common association of pincer nails with ingrown nails (Fig. 5). First, genetic predisposition and/or systemic diseases may result in a naturally excessive automatic shrinkage force that causes the nail to curve inward. When these nails are cut to the quick, they stick into the lateral soft tissue, which leads to pain and inflammation. The patient then seeks to reduce the pain-inducing load on the nail by altering the gait, reducing ambulation, and/or wearing ill-fitting shoes, which in turn further reduces the upward mechanical force on the first toe. This vicious circle leads to further overcurvature of the nail. Ill-fitting shoes and a bedridden condition may also lead to such overcurvature. Thus, risk factors for pincer nail may be hereditary, systemic diseases, wearing of ill-fitting shoes, a bedridden state and inappropriate nail cutting, as these factors contribute to nail overcurvature either constitutively or by reducing/abrogating the mechanical forces on the toe nail. This hypothesis has practical implications as it suggests that the pincer nails of walkers may be treated by correcting their walking behavior so that more pressure is applied onto the toe pad.

## Conclusion

The gait characteristics of cases of pincer nail were assessed in this study. The results suggested that walking behavior may contribute to the development of pincer nail.
